# Whether intermediate-risk stage 1A, grade 1/2, endometrioid endometrial cancer patients with lesions larger than 2 cm warrant lymph node dissection?

**DOI:** 10.1186/s12885-017-3671-0

**Published:** 2017-10-23

**Authors:** Menghan Zhu, Nan Jia, Feifei Huang, Xiaoxia Liu, Yuqing Zhao, Xiang Tao, Wei Jiang, Qin Li, Weiwei Feng

**Affiliations:** 1 Department of Gynecology, Obstetrics and Gynecology Hospital, Fudan University, Shenyang Road 128, Shanghai, 200090 China; 2 Department of Pathology, Obstetrics and Gynecology Hospital, Fudan University, Shenyang Road 128, Shanghai, 200090 China; 3 Shanghai Key Laboratory of Female Reproductive Endocrine-Related Disease, Fudan University, Zhaozhou Road 413, Shanghai, 200011 China; 4 Present Address: Department of Gynecology and Obstetrics, Ruijin Hospital, Shanghai Jiaotong University, School of Medicine, 197 Ruijin Road, Shanghai, 200025 China

**Keywords:** Endometrial cancer, Endometrioid, Intermediate-risk, Lymphadenectomy, Prognosis

## Abstract

**Background:**

Our research aimed to investigate whether lymphadenectomy was required in patients with intermediate-risk endometrioid endometrial cancer (EEC).

**Methods:**

Between 1989 and 2015, 1009 patients with intermediate-risk EEC: grade 1 or 2 tumor, <50% myometrial invasion, and a tumor diameter ≥ 2 cm and 818 low-risk patients with grade 1 or 2 tumor, <50% myometrial invasion, and a tumor diameter < 2 cm were enrolled in this study. The rate and risk factors of node metastasis were evaluated and compared between the two risk groups. Survival data were analyzed in patients with intermediate-risk EEC with or without lymphadenectomy.

**Results:**

In all, 624 of 1009 (61.8%) patients with intermediate-risk EEC underwent pelvic ± para-aortic lymphadenectomy with a nodal metastasis rate of 1.9% (12/624), whereas 394 of 818 (48.2%) patients with low-risk EEC underwent pelvic ± para-aortic lymphadenectomy with a nodal metastasis rate of 0.3% (1/394) (*p* = 0.021). Notably, intermediate-risk EEC patients with a microcystic, elongated and fragmented (MELF) pattern of invasion, lymphatic vascular space invasion (LVSI), diffuse lesions, or lesions located in the cornua were more likely to have node metastasis. The 5-year overall cancer-related survival and the recurrence-free survival rates of the 742 intermediate-risk EEC patients who were followed for more than 3 years were 99.4% and 94.7%, respectively. In intermediate-risk group, 6 patients (6/443, 1.4%) with lymphadenectomy and 9 patients (9/299, 3.0%) without lymphadenectomy recurred, with a mean recurrence time of 38.3 and 18.7 months respectively. The five-year overall and recurrence-free survival rates of intermediate-risk patients with and without lymphadenectomy were similar (100% vs 98.9%, *p* = 0.351; 95.2% vs 93.3%, *p* = 0.464).

**Conclusion:**

Patients with intermediate-risk EEC have low nodal metastasis rate and a favorable outcome whether lymphadenectomy is performed or not. Omission of lymphadenectomy may be a reasonable option in the surgical management of patients with intermediate-risk EEC.

## Background

Worldwide, endometrial cancer is one of the most common malignant tumors of the female reproductive system and is a major cause of mortality for patients. Total hysterectomy associated with bilateral salpingo-oophorectomy (TH/BSO) is a standard component of endometrial cancer treatment, especially for early-stage endometrial cancer [1]. Since 1988, the International Federation of Obstetrics and Gynecology (FIGO) has recommended surgical staging for endometrial cancer patients. Lymph node involvement is the most important prognostic factor for early clinical stage disease [2].

However, surgical assessment of lymph nodes for staging during primary surgery remains one of the most varied practices worldwide, as it may include no nodal assessment, sentinel node mapping, and complete pelvic and aortic lymphadenectomy up to the renal vessels. Since lymphadenectomy is significantly associated with longer operating time, higher surgical costs, greater rate of infection, as well as the occurrence of lymphocysts and lymphedema [3, 4], gynecologists agree that pelvic and aortic lymphadenectomy should be routinely performed in high-risk patients (grade 3, deep myometrial invasion, type 2 cancer). However, whether lymphadenectomy is required in patients with endometrioid endometrial cancers of grade 1 or 2 and with less than 50% myometrial invasion is controversial [5–8].

Many studies have been conducted to investigate prognostic factors that identify risk stratification systems and have attempted to determine the optimal choices for surgical and adjuvant treatment. Epidemiological and histological factors such as increasing age, depth of myometrial invasion, histological tumor type and grade, tumor size, and the presence of lymphatic vascular space invasion (LVSI) are among the possible predictors of a higher risk of recurrence and nodal metastasis [9–14]. Adenocarcinomas of grade 1 or 2 and those with less than 50% myometrial invasion are usually defined as low risk or intermediate risk, and thus different treatments are tailored to different tumors. For example, the International Federation of Gynecology and Obstetrics (FIGO 2015) and the European Society for Medical Oncology (ESMO) (2016) define all patients with stage IA and grade 1 or 2 endometrioid cancer regardless of the tumor size as low-risk and systematic lymphadenectomy is not recommended in these patients [15, 16]. However, the National Comprehensive Cancer Network (NCCN) (2016) define patients with endometrioid, grade 1 or 2, myometrial invasion ≤50%, and a primary tumor diameter ≤ 2 cm as low-risk and systematic lymphadenectomy may not require in these patients [17]. In addition, NCCN consider patients with deeply invasive lesions, high-grade histology, and tumors of serous carcinoma, clear cell carcinoma, or carcinosarcoma features as high risk. Thus there is a gap between low-risk and high-risk patients. This subgroup of patients are grade 1 or 2 endometrioid carcinoma, less than 50% myometrial invasion and tumor diameter > 2 cm. In this study, we define this subgroup as intermediate-risk patients.

The main controversy is whether to perform lymphadenectomy, and if it is performed, to what extent this procedure should be performed (e.g., pelvic nodes only, or both pelvic and aortic nodes) in this subgroup patients. To address this concern, accurate data on the rates of lymph node metastasis are required.

In 2000, the Mayo clinic reported that, out of 59 patients who met the criteria for low-risk endometrioid endometrial cancer (tumor size ≤2 cm, histologic grade 1 or 2, and ≤50% myometrial invasion), no patient had detectable lymphatic dissemination [10]. In a 2012 multicenter post hoc analysis, the author used the modified Mayo Criteria (tumor size <2 cm, histologic grade 1 or 2, and <50% myometrial invasion) to define low-risk endometrioid endometrial cancer [17]. The study indicated a rate of 0.8% (3/389) for nodal metastasis in the low-risk group. However, data on the nodal metastasis rate are rare among patients with intermediate-risk EEC (grade 1 and 2, <50% myometrial invasion and a lesion ≥2 cm). In a study published in 2000 by the Mayo Clinic, which is mentioned above, 8 of 107 patients with a primary tumor diameter > 2 cm (7%) had pelvic lymph node dissemination, but all of them demonstrated >50% myometrial invasion.

Hence, we conducted this retrospective study to investigate the characteristics of patients with intermediate-risk EEC (grade 1 or 2, <50% myometrial invasion, and a tumor diameter ≥ 2 cm) as well as their outcomes. We aimed to determine the rate of nodal metastasis, to distinguish factors that influence lymph node involvement and to provide prognostic significance for performing lymphadenectomy in this group of patients.

## Methods

We reviewed the medical records of patients with endometrial cancer who underwent surgical treatment for the disease at the Obstetrics and Gynecology Hospital of Fudan University between 1989 and 2015. The data in the study were collected from the hospital’s archived database and were used only for research. The study was approved by the ethics committee of the Obstetrics and Gynecology Hospital of Fudan University.

In all, 1009 patients with intermediate-risk and 614 low-risk EEC were enrolled in this study regardless of lymph node status. Since specimens from a certain number of patients were not subjected to comprehensive intraoperative frozen section assessment, we were unable to stratify the risk group based on the frozen section reports. Patients were identified as intermediate risk based on three specific criteria within the final pathology reports: 1) histologic grade 1 or 2; 2) <50% myometrial invasion; and 3) and tumor diameter ≥ 2 cm. Patients who met these criteria with the exception of a tumor diameter less than 2 cm were classified into the low-risk group. TH/BSO was the standard procedure for these patients, and all surgeries were performed by senior gynecologic oncologists. Lymphadenectomy was performed if the surgeon considered the patient to be at risk for nodal metastasis, which was determined by characteristics of the patient such as age, history and general condition. Pelvic node-bearing tissues in the external iliac, internal iliac, obturator, and common iliac regions were removed. Para-aortic lymph node dissection covered the inferior mesenteric artery region. Whether laparotomy or laparoscopic surgery was performed was based on surgeon preference.

**Fig. 1:**
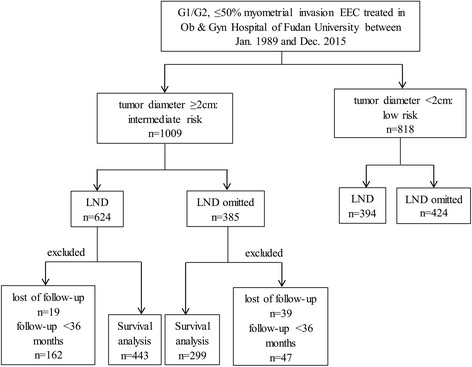
Diagram of the study design

Information that was collected included age, preoperative CA125 level, details regarding the surgical specimens (e.g., tumor diameter, lesion location, number of excised lymph nodes) and histologic reports of the specimens (e.g., tumor subtype, tumor grade, uninvolved endometrium, depth of myometrial invasion, LVSI, lymph node status and follow-up outcome). Seven hundred and forty-two patients who were followed-up for at least 3 years and those who met the end point were subjected to survival analysis. The process of this analysis is shown in Fig. 1.

Before fixation, each uterus was incised through the anterior midline in order to open the cavity and to orient the tissue. The size of the lesion was measured to determine its largest three dimensions. The tumor diameter was defined as the largest diameter of the three dimensions. The locations of the lesions were subdivided into the following categories: fundus, cornua, side wall, lower uterine segment and diffuse lesion.

The histologic types of the tumors and the uninvolved endometrium, as well as the histologic tumor grade, were diagnosed according to the World Health Organization Classification of Tumors: Pathology and Genetics of Tumors of the Breast and Female Genital Organs (2014).

In hematoxylin and eosin-stained (H&E) sections, LVSI was considered to be present when tumor cells were observed within or attached to the wall of a blood vessel or within the lymphatic space. In immunohistochemical sections stained for CD31, LVSI was recorded when any number of tumor cells was observed within the lymphatic vascular space, and recorded as positive when it presented on either H&E-stained or CD31-immunostained sections.

All the H&E-stained and immunostained slides containing the surgical specimen were reviewed retrospectively by two experienced gynecological pathologists for confirmation of the original diagnoses.

Analysis of the data was performed with IBM© SPSS® 20 software for Windows (SPSS Inc., Chicago, IL, USA). Pearson χ2 tests and t-tests were utilized to identify the differences in clinicopathological characteristics between intermediate-risk and low-risk patients. Fisher’s exact tests and t-tests were utilized to identify factors that were related to the presence of nodal metastasis according to the univariate analysis. The results were considered statistically significant at *p* < 0.05. The overall 5-year survival and recurrence-free survival rates were determined using the Kaplan-Meier method and were compared using the Log-rank test. To determine the outcome of patients who experienced recurrence, patients who did not experience recurrence were treated as censored observations. To determine the rate of cancer-related death, patients who were alive at the end of the follow-up period and those who died from a cause other than cancer were treated as censored observations.

## Results

### The rate of node metastasis in patients with intermediate-risk and low-risk EEC

Among the 1827 patients, 1009 were identified as intermediate risk, while 818 were deemed to be low risk. The patient characteristics are shown in Table 1. The serum CA125 level in all 1827 patients was mostly within the normal range, but nevertheless, it was higher in the intermediate-risk group than in the low-risk group (23.36 ± 32.67 vs. 18.08 ± 22.43, *p* = 0.023). Histologic grade 2 tumors and LVSI presented more frequently in the intermediate-risk group than in the low-risk group (18.4% vs. 7.5%, *p* < 0.001; 4.7% vs 2.2%, *p* = 0.005). More patients in the low-risk group had lesions that were confined to the endometrium with a rate of 41.4% in comparison with 13.4% in patients in the intermediate-risk group (*p* < 0.001). Other factors, including age, medical history (e.g., hypertension, diabetes, family history of malignant tumors), and histologic tumor subtype (endometrioid or areas of squamous differentiation), were not different between the two groups.

**Table 1:** Characteristics of low-risk and intermediate-risk endometrioid endometrial cancer patients

		Low risk (*n* = 818)	Intermediate risk (*n* = 1009)	p
Age(years)		53.0 ± 8.5(27–79)	53.2 ± 9.0(26–84)	0.663^a^
Hypertension	yes	175(21.4%)	249(24.7%)	0.106^b^
	no	643(78.6%)	760(75.3%)	
Diabetes	yes	67(8.2%)	82(8.1%)	1.000^b^
	no	751(91.8%)	927(91.9%)	
Family history of malignant tumor	yes	77(9.4%)	73(7.2%)	0.103^b^
	no	741(90.6%)	936(92.8%)	
Serum CA125 level		18.08 ± 22.43	23.36 ± 32.67	0.023^a^
Histologic subtype	Endometrioid	784(95.8%)	971(96.2%)	0.717^b^
	Areas of squamous differentiation	34(4.2%)	38(3.8%)	
Tumor grade	1	757(92.5%)	823(81.6%)	<0.001^b^
	2	61(7.5%)	186(18.4%)	
Myometrial invasion	none	339(41.4%)	135(13.4%)	<0.001^b^
	Superficial (<50%)	479(58.6%)	874(86.6%)	
LVSI	yes	18(2.2%)	47(4.7%)	0.005^b^
	no	800(97.8%)	962(95.3%)	
Lymphadenectomy	yes	394(48.2%)	624(61.8%)	***<***0.001^b^
	no	424(51.8%)	385(38.2%)	

^a^t test

^b^Pearson χ2 test

In the intermediate-risk group, 385 (38.2%) patients underwent TH/BSO only, while 624 (61.8%) underwent TH/BSO and lymphadenectomy simultaneously. Of these 624 patients, 520 (83.3%) underwent pelvic lymphadenectomy only, and 104 (16.7%) underwent pelvic and para-aortic lymphadenectomy. The median number lymph nodes harvested were 20 (range, 2–52) and 20(range, 1–44)in intermediate-risk and low-risk patients. The median number para-aortic nodes harvested were 5 (range 1–23) and 4(range 1–15)in intermediate-risk and low-risk patients. Twelve (1.9%; 95% CI, 0.8%–3.0%) patients were found to have node metastasis, and in 2 of these 12 patients, the para-aortic lymph nodes were involved. In the low-risk group, 394 (48.2%) patients underwent lymphadenectomy, among which, 341 underwent pelvic lymphadenectomy only, and 53 underwent pelvic and para-aortic lymphadenectomy. One patient had pelvic node metastasis. The rate of nodal metastasis was significantly different between the patients with intermediate-risk and low-risk EEC (1.9% vs. 0.3%, *p* = 0.021). The median number of harvested pelvic and para-aortic lymph nodes was similar in the two risk groups.

### Risk factors for node metastasis in patients with intermediate-risk EEC

We further observed the characteristics of patients with intermediate-risk EEC with and without nodal metastasis (Table 2). Among all 624 intermediate-risk patients who underwent lymphadenectomy, a microcystic, elongated and fragmented pattern (MELF) of invasion was observed in 7 patients, and 2 (28.6%) of these patients had node metastasis. The metastasis rate in patients with and without LVSI was 11.8% (4/34) and 1.4% (8/590), respectively (*p* < 0.05). With regard to lesion location, it seemed that lesions located in the cornua (3/87, 3.4%) and diffuse lesions (6/126, 4.8%) were more likely to metastasize than lesions in the side wall and lower segment (1/213, 0.5%) and those in the fundus (2/198, 1.0%).

**Table 2:** Factors related to node metastasis in intermediate-risk EEC patients who underwent lymphadenectomy

		Total(*n* = 624)	Node metastasis (*n* = 12)	p
Histologic subtype	endometrioid	601	11 (1.8%)	0.372^a^
	areas of squamous differentiation	23	1 (4.3%)	
Tumor grade	1	504	8 (1.6%)	0.263^a^
	2	120	4 (3.3%)	
Histologic type of uninvolved endometrium	simple hyperplasia	153	3 (2.0%)	0.636^a^
	complex hyperplasia	5	0	
	secretory endometrium	19	0	
	complex hyperplasia with atypia	143	1(0.7%)	
	atrophic endometrium	121	2 (1.7%)	
	no uninvolved endometrium	183	6 (3.3%)	
Myometrial invasion	none	65	0	0.004^a^
	superficial(<50%)	552	10 (1.8%)	
	superficial with MELF	7	2 (28.6%)	
LVSI	no	590	8 (1.4%)	0.004^a^
	yes	34	4 (11.8%)	
Lesion location	fundus	198	2 (1.0%)	0.020^a^
	cornua	87	3 (3.4%)	
	diffuse lesion	126	6(4.8%)	
	side wall & lower uterine segment	213	1(0.5%)	

Data anaylysis

^a^Fisher exact test

The detailed characteristics of the 12 intermediate-risk patients with nodal metastasis are listed in Table 3. Para-aortic lymphadenectomy was performed in 4 patients. Nodal metastasis primarily involved the pelvic lymph nodes, while one patient had only para-aortic lymph node involvement and another had both pelvic and para-aortic lymph node involvement. Of the 12 patients with nodal metastasis, all lesions had invaded the myometrium.

**Table 3:** Characteristics of the 12 intermediate-risk EEC patients with nodal metastasis

No.	Age range (years)	Tumor grade	Myometrial invasion	LVSI	Tumor maximun diameter(mm)	Lesion location	Para-aortic LND	Location of involved lymph node	Observation time(months)	Adjuvant treatment^b^	Outcome
1	60–65	G1	<50%	no	80	diffuse lesion	no	right obturator 1/7	94	refused	NED
2	55–60	G1	<50%	no	62	diffuse lesion	no	left iliac vessel 1/5	42	refused	NED
3	50–55	G2	<50%	no	24	Side wall	no	right obturator 1/2	124	chemotherapy + radiotherapy	NED
4	60–65	G2	<50%	no	45	diffuse lesion	no	right obturator 1/3	61	radiotherapy	NED
5^a^	55–60	G2	<50%	no	30	fundus	yes	left obturator 1/4	35	chemotherapy	NED
6	60–65	G1	<50%	yes	50	diffuse lesion	no	right iliac vessel 1/6	36	chemotherapy	NED
7	40–45	G1	<50%	no	20	right cornua	no	right obturator 1/1	38	refused	NED
8^a^	60–65	G2	<50%	yes	35	left cornua	yes	solely para-aortic 1/6	28	refused	NED
9^a^	50–55	G1	<50%	no	22	right cornua	no	left iliac vessel 1/5	12	radiotherapy	NED
10^a^	45–50	G1	<50%	no	60	diffuse lesion	yes	left iliac vessel 2/8	24	refused	NED
11^a^	50–55	G1	<50% with MELF	yes	40	fundus	no	left iliac vessel 1/9	12	refused	NED
12^a^	45–50	G1	<50% with MELF	yes	75	diffuse lesion	yes	left iliac vessel 1/10,right iliac vessel 1/10, para-aortic 1/7	12	chemotherapy + radiotherapy	NED

*LND* lymph node dissection

*NED* no evidence of disease

^a^The surgery dates of these 12 patients range from October 1999 to December 2015, thus 6 patients were observed less than 36 months postoperatively

^b^Patients who received chemotherapy were all treated with paclitaxel and carboplatin for 6 cycles. Patients underwent radiotherapy were given external- beam radiation therapy (EBRT) as 5040 cGy given as 28 fractions of 180 cGy

### Patients with intermediate-risk EEC had a favorable outcome

We further analyzed the survival data of the intermediate-risk patients. The median follow-up time was 51 months (range, 4–174 months). Patients followed-up ≥36 months were included for survival analysis. In all, 209 patients who underwent surgery from 2013 to 2015 and were followed-up for <36 months were excluded from the survival and recurrence analysis. In addition, these 209 patients were all alive with no evidence of cancer recurrence during the observation period. Fifty-eight patients were lost to follow-up. In all, 742 patients were enrolled in the survival and recurrence analysis. All censored cases had a follow-up time ≥ 36 months except those who died from other causes. The median follow-up time was 65 months (range, 36–174 months). The 5-year overall cancer-related survival of all intermediate-risk patients was 99.4%, and the 5-year recurrence-free survival was 94.7%. During our observation period, 716 patients (96.5%) were alive without evidence of disease, 9 (1.2%) was alive with disease, 6(0.8%)died of recurrence, and 11(1.5%) patients died of a cause other than cancer. Overall, 15 incidents of recurrence (2.0%) were observed. The median time to recurrence was 23 months (range, 5–62 months). Five patients had local recurrences, and all of them were alive at the end of follow-up. Eight patients had distant recurrences and 4 of them had died by the end of follow-up. The 2 patients who experienced recurrence at both local and distant sites also died. Of the 10 instances of recurrence at distant sites, 3 involved the lung. All 15 patients with cancer recurrence were free of LVSI. In addition, we were unable to determine the different characteristics between patients who experienced recurrence and those who did not based on our clinical data.

The 12 patients with lymph node metastasis are now all alive with no evidence of disease progression. Six of them, however, underwent adjuvant therapy after their initial surgery.

### Lymphadenectomy had no impact on the survival of intermediate-risk patients

**Fig. 2:**
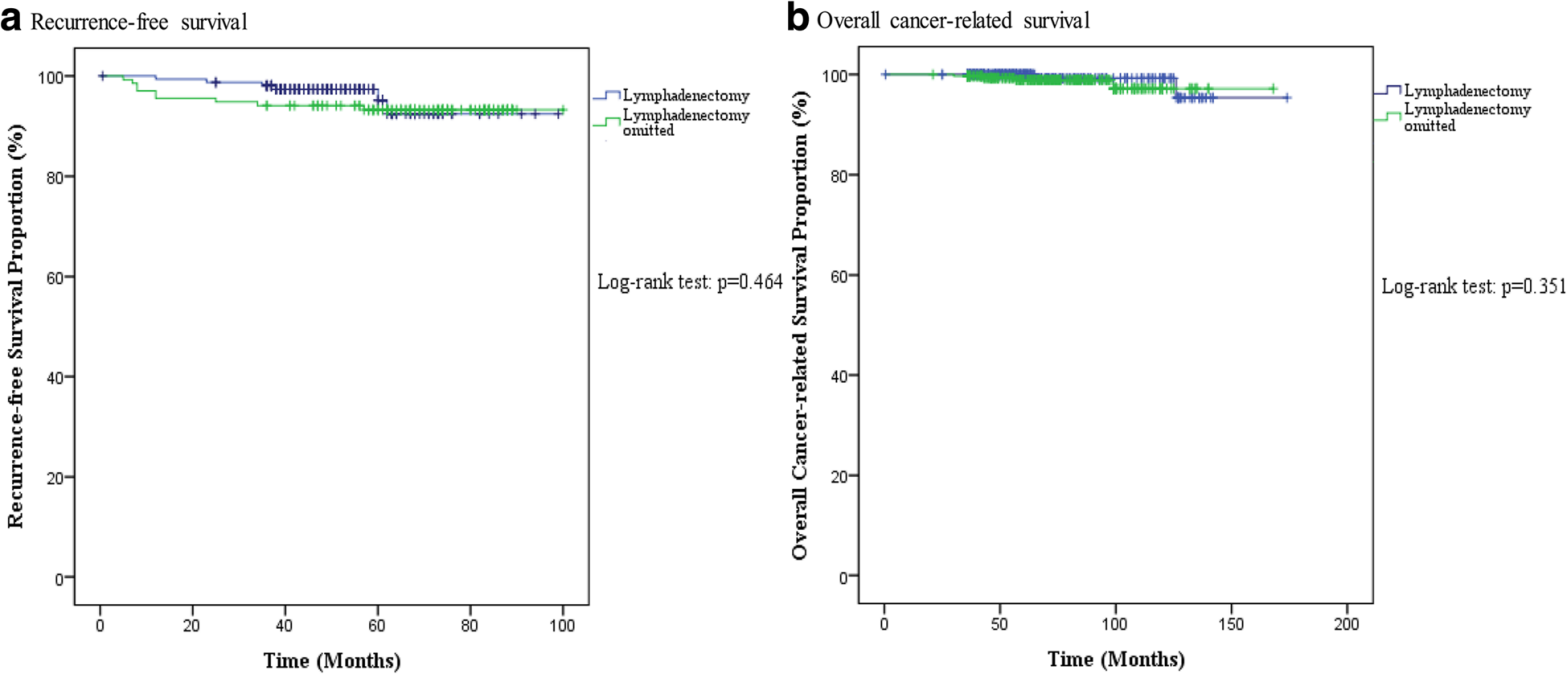
Survival analysis for intermediate-risk EEC patients with and without lymphadenectomy. Recurrence –free survival (**a**) and overall cancer-related survival (**b**) were not different between intermediate-risk EEC patients with and without lymphadenectomy

Subsequently, we summarized the clinicopathological features of the 15 patients with recurrence including 6 who received lymphadenectomy and 9 who did not (Table 4). The 6 patients had no node metastasis at the time of the initial surgery. The recurrence rates of the intermediate-risk patients who underwent lymphadenectomy and those who did not were both minimal. Six patients with intermediate-risk EEC who underwent lymphadenectomy (6/443, 1.4%) experienced recurrent cancer with a mean time to recurrence of 38.3 months. Meanwhile, 9 intermediate-risk patients who did not undergo lymphadenectomy (9/299, 3.0%) experienced recurrent cancer with a mean time to recurrence of 18.7 months. The recurrence interval of intermediate-risk patients who underwent lymphadenectomy was significantly short than patients who did not undergo lymphadenectomy (*p* < 0.05, Mann–Whitney Test), however, the 5-year recurrence-free survival showed no significant difference between these subgroups. Among the intermediate-risk patients who underwent lymphadenectomy, the 5-year overall cancer-related survival was 100%, while the 5-year recurrence-free survival was 95.2%. Among the intermediate-risk patients who did not undergo lymphadenectomy, the 5-year overall cancer-related survival was 98.9%, and the 5-year recurrence-free survival was 93.3%. Lymphadenectomy was not associated with a longer recurrence-free survival (*p* > 0.05) or overall cancer-related survival (*p* > 0.05) (Fig. 2).

**Table 4:** Characteristics of the 15 intermediate-risk EEC patients with recurrence

Group	Age range (years)	Tumor grade	Myometrial invasion	Tumor maximum diameter (mm)	Lesion location	Treatment after surgery	Interval between initial surgery and recurrence(months)		Site of recurrence	Treatment after recurrence	Outcome	Observation time (months)
	50–55	G1	<50%	20	fundus	none	60	distant(lung involved)	chemotherapy	alive		75
	70–75	G2	<50%	40	diffuse lesion	none	35	distant(lung involved)	palliative treatment	died of recurrence		66
LND	50–55	G1	<50%	20	side wall	none	12	local	chemotherapy + radiotherapy	alive		20
*n* = 6	65–70	G1	<50%	30	left cornua	none	38	local	traditional Chinese medicine	alive		41
	65–70	G2	<50%	20	side wall	none	62	local + distant	chemotherapy	died of recurrence		126
	60–65	G1	<50%	26	left cornua	none	23	local	radiotherapy	alive		48
	65–70	G2	<50%	25	side wall	none	57	local + distant	unknown	died of recurrence		57
	50–55	G1	<50%	25	side wall	chemotherapy + radiotherapy	5	local	radiotherapy	alive		85
	50–55	G1	<50%	32	side wall	none	25	local	radiotherapy	alive		66
LND omitted	65–70	G1	<50%	24	fundus	none	34	distant	surgery	alive		47
*n* = 9	55–60	G1	<50%	20	left cornua	none	8	local	surgery + chemotherapy	alive		76
	55–60	G1	<50%	50	side wall	chemotherapy	12	distant(lung involved)	chemotherapy	alive		49
	45–50	G1	<50%	22	lower uterine segment	chemotherapy	12	distant	chemotherapy	died of recurrence		99
	65–70	G2	<50%	30	side wall	none	8	distant	chemotherapy	died of recurrence		43
	65–70	G2	none	50	right cornua	none	7	distant	chemotherapy	died of recurrence		30

*LND* lymph node dissection

## Discussion

Whether lymphadenectomy is required for early-stage endometrioid endometrial cancer remains controversial. Current guidelines have reached a consensus that lymphadenectomy is not necessary for patients with low-risk EEC. However, in these guidelines, the criteria that define “low risk” are different (Table 5). The main discrepancy is whether patients with lesions larger than 2 cm should be classified as low-risk and undergo lymphadenectomy. In our study, we investigated EEC patients with tumors of histologic grade 1 or 2 and <50% myometrial invasion. We classified patients with tumors <2 cm in diameter as low risk and those with tumors ≥2 cm in diameter as intermediate risk, and then carefully evaluated the node metastasis rates and related factors in both groups. We found that the risk of nodal metastasis for intermediate-risk endometrial cancer was low (1.9%). Further analysis of related factors indicated that MELF invasion, LVSI, and lesion location may correlate with nodal metastasis.

**Table 5:** Recommendation for lymphadenectomy in endometrial cancer in clinical practice

Guideline	Low-risk	Intermediate risk	High risk	Lymphadenectomy required for low-risk patients
FIGO (2015) [16]	Grade 1 or grade2, < 1/2 myometrial invasion	/	grade 3,>50% of myometrial invasion,lymphovascular space invasion,non-endometrioid histology (serous, clear cell, undifferentiated, small cell, anaplastic, etc). cervical stromal involvement	systematic lymphadenectomy is not recommended,suspicious lymph nodes sampling is recommended
ESMO(2016) [15]	grade 1 or 2 and superficial myometrial invasion <50%	grade 1 or 2 deep myometrial invasion >50% or grade 3 superficial myometrial invasion <50%	grade 3 with deep myometrial invasion >50%	systematic lymphadenectomy is not recommended for LR patients (it can be considered for IR patients)
NCCN(NCCN Guidelines®, uterine neoplasms, version 2. 2016)	less than 50% myometrium invasion, tumor diameter < 2 cm, well or moderate differentiated histology	/	deeply invasive lesions, high-grade histology, and tumors of serous carcinoma, clear cell carcinoma, or carcinosarcoma features	systematic lymphadenectomy is not necessary.Excision suspious or enlarged lymph nodes is recommended

In our study, patients with low-risk EEC were also more likely to have a low rate of nodal metastasis (0.3%), which is consistent with the results of other studies [10, 17–20].

However, for patients in accordance with the criteria that defined intermediate risk in our study, neither node involvement data nor prognosis data have been reported. Our study is the first that is specifically focused on this subset of patients. We found that the nodal metastasis rate was low in patients with intermediate-risk EEC, although it was higher than that in patients with low-risk EEC. The prognosis of patients with intermediate-risk EEC indicated no difference in overall cancer-related survival or recurrence-free survival whether lymphadenectomy was performed or not.

According to the Mayo Criteria, tumor size, which is defined by a 2-cm threshold, is a decisional index for risk stratification. We stratified each of the risk groups based on tumor size using a cut-off value of 2 cm. One case of lymph node involvement was found in the low-risk group, while a rate of 1.9% nodal metastasis was reported in the intermediate-risk group. A French multicenter study demonstrated that tumor size is an additional prognostic factor of lymph node involvement in women with low-risk endometrial cancer [21]. According to their study, a tumor size ≥35 mm emerged as the optimal threshold for a higher rate of nodal involvement. Turan et al. reported that tumor size was an independent prognostic factor for pelvic lymph node metastasis, and the cut-off was determined to be 40 mm [22]. In our study, the mean tumor diameter in patients with intermediate-risk EEC with nodal metastasis was 45.25 ± 20.47 mm. However, no significant difference was found between patients with intermediate-risk EEC with and without nodal metastasis (37.70 ± 20.38 vs. 45.25 ± 20.47, *p* > 0.05) (data not shown).

Lymphatic vascular space invasion has been identified as a potential adverse risk factor for nodal metastasis and prognosis [14, 19, 23–27]. In our study, positive LVSI was more frequently found in patients with intermediate-risk EEC than in those with low-risk EEC (4.7% vs. 2.2%). Moreover, the nodal metastatic rate in patients with intermediate-risk EEC with LVSI was much higher than that in patients without LVSI (11.8% vs. 1.4%, *p* < 0.05). Dos Reis et al. demonstrated that patients with low-risk endometrial cancer (grade 1 or 2 endometrioid histology, myometrial invasion <50%, and disease confined to the uterus) and LVSI had a worse relapse-free survival and overall survival even though they were more likely to undergo lymphadenectomy and adjuvant therapy [19]. According to our survival and recurrence analysis, however, all 15 patients with cancer recurrence were free of LVSI. Furthermore, LVSI is difficult to assess using intraoperative frozen sections, and it is often reported in the final pathology report. Thus, despite the finding that positive LVSI was associated with lymph node involvement, lymphadenectomy performed during the initial surgery, as indicated by the presence of LVSI, is very difficult.

Myometrial invasion is another well-defined risk factor for endometrial cancer [9, 11, 12, 14]. Deep invasion into the myometrial wall is correlated with poor prognosis and lymph node metastasis. The patients enrolled in our study were diagnosed with less than 50% myometrial invasion, and some of these patients merely had disease that was confined to the endometrium. Of the patients with nodal metastasis, all invasion was limited to the superficial myometrium. Several studies have indicated that an MELF pattern of invasion is associated with an increased rate of lymph node involvement and is a significant predictor of nodal metastasis [24, 28]. In our study, 2 of 7 patients who were diagnosed with intermediate-risk EEC with an MELF pattern of invasion had node metastasis (28.6%). Myometrial invasion with an MELF pattern of invasion can be subtle, and failure to detect it may lead to an underestimation of the depth of myometrial invasion, which may result in tumor under-staging [29, 30]. Hence, this may explain the correlation between an MELF pattern of myometrial invasion and lymph node metastasis.

The location of the tumor within the uterus is also an important indicator. Involvement of the uterine isthmus, cervix, or both is associated with an increased risk for extrauterine disease and lymph node metastasis as well as recurrence [13, 31, 32]. In our study, patients with intermediate-risk EEC with nodal metastasis were more likely to have lesions located in the cornua (3/87, 3.4%) and were more likely to have diffuse lesions (6/126, 4.8%).

We analyzed the prognosis of patients with intermediate-risk EEC who underwent lymphadenectomy or not. The results showed that all patients with intermediate-risk ECC exhibited good overall survival and recurrence-free survival. The 12 patients with lymph node metastasis are now all alive with no evidence of disease progression. Six of them received adjuvant therapy after initial surgery. As a consequence, although these patients already have nodal metastasis, good disease control may result from adjuvant therapy.

Notably, among the 15 cases of recurrent intermediate-risk EEC without node metastasis or assumed node metastasis (lymphadenectomy not performed), 10 patients had recurrences at distant sites, including 3 in the lung. This may indicate that early-stage EEC might metastasize through the blood with a greater likelihood than was previously thought. However, those patients were also free of LVSI. In addition, we were unable to determine the different characteristics between patients who experienced recurrence and those who did not. Moreover, lymphadenectomy was found to be unrelated to overall cancer-related survival and recurrence-free survival in patients with intermediate-risk EEC. Recurrence cannot be interpreted using only pathological prognosticators and surgical procedures. Molecular genetic features might be drivers of recurrence and should thus be further investigated as they may be able to guide post-surgical treatment.

Considering no difference was found in overall cancer-related survival or recurrence-free survival whether lymphadenectomy was performed or not, we assume that lymphadenectomy may not be compulsory in the treatment of patients with intermediate-risk EEC. Although our study revealed that an MELF pattern of invasion, LVSI and specific lesion locations were possible factors related to nodal metastasis, the intra-operative decision for lymphadenectomy relies only on the lesion location, since the other information (MELF invasion and LVSI) is typically only reported in the final pathology reports. Thus, a certain proportion of patients with intermediate-risk EEC still underwent unnecessary lymphadenectomy. In clinical practice, sentinel lymph node mapping might be a solution that can be used to identify the patients who indeed require lymphadenectomy. On the contrary, molecular diagnostic features of curettage samples can be developed to predict node metastasis and can be used for guidance of lymphadenectomy.

## Conclusions

In summary, the risk of nodal metastasis is higher in patients with intermediate-risk EEC than in patients with low-risk EEC, although it is considerably low in both groups. An MELF-pattern of invasion, LVSI and specific lesion locations are factors that may be related to nodal metastasis in patients with intermediate-risk EEC. Patients with intermediate-risk EEC have a favorable outcome regardless of whether lymphadenectomy is performed or not. The omission of lymphadenectomy may be a reasonable option in the surgical management of those patients with intermediate-risk EEC.

One weakness of our study is its retrospective nature and the lack of preoperative or intra-operative assessment. Our nodal metastasis rate may be underestimated, as not all the patients with intermediate-risk EEC underwent LND. Tumor size was assessed in uterine specimens, but the potential impact of the preoperative biopsy on tumor size was not considered. Further studies that include a preoperative imaging examination of the tumor volume as a decisional criterion may be required. The strengths of our study are the large size of the study population, the extensive investigation of variables and the preferred follow-up time. We are currently in the process of continuing to follow-up all of these patients to supplement our results.

## Abbreviations

EEC: Endometrioid endometrial cancer
LVSI: Lymphatic vascular space invasion
MELF: Microcystic, elongated and fragmented
FIGO: The International Federation of Obstetrics and Gynecology
ESMO: The European Society for Medical Oncology
NCCN: The National Comprehensive Cancer Network
CI: Confidence interval
CA 125: Carbohydrate antigen 125
